# The Effects of Displacing Sedentary Behavior With Two Distinct Patterns of Light Activity on Health Outcomes in Older Adults (Implications for COVID-19 Quarantine)

**DOI:** 10.3389/fphys.2020.574595

**Published:** 2020-12-11

**Authors:** Dale Grant, David Tomlinson, Kostas Tsintzas, Petra Kolić, Gladys Leopoldine Onambele-Pearson

**Affiliations:** ^1^Department of Sports and Exercise Sciences, Research Centre for Musculoskeletal Science and Sports Medicine, Manchester Metropolitan University, Manchester, United Kingdom; ^2^MRC Versus Arthritis Centre for Musculoskeletal Ageing Research, Faculty of Medicine & Health Sciences, School of Life Sciences, The University of Nottingham Medical School, Queen’s Medical Centre, Nottingham, United Kingdom

**Keywords:** COVID-19, physical functioning, sedentary behaviour, sit-to-stand, triglyceride, light intensity physical activity

## Abstract

**Rationale:** The COVID-19 pandemic is limiting outdoor and community-based activities, especially for older adults owing to the requirement for self-isolation, potentially increasing prolonged sedentary behavior (SB). Given a poor tolerance for intense exercise, SB displacement with light intensity physical activity (LIPA) is a promising health enhancing alternative. Therefore, the aims of this study were to investigate the effects of two different types of SB displacement on health outcomes in older adults and any differential impact of associated LIPA pattern.

**Method:** 28 older women (age: 73 ± 5 years, height: 1.60 ± 0.07 m, weight: 67 ± 10 kg, and BMI: 26.1 ± 3.6 kg/m^2^) underwent overnight fasted dual energy x-ray absorptiometry (DEXA) imaging, blood sampling, and functional assessments before being randomly allocated to one of two groups: (1) single continuous bout of 45–50 min LIPA daily (*n* = 14); or (2) SB fragmentation (SBF; ~48 min LIPA daily, 2 min LIPA for every 30 min of SB; *n* = 14). Compliance was systematically monitored using tri-axial accelerometery. All measures were taken at weeks 0 and 8.

**Results:** Physical behavior significantly altered (decreased SB/increased LIPA; *p* < 0.05) and to a similar extent in both groups. We observed a significant reduction in serum triglycerides [*p* = 0.045, effect size (*ɳ_p_*^2^) = 0.15; SBF: −0.26 ± 0.77 mmol/L, LIPA: −0.26 ± 0.51 mmol/L], improved 30 s sit-to-stand (STS) count (*p* = 0.002, *ɳ_p_*^2^ = 0.32, 2 ± 3 STS) and speed (*p* = 0.009, *ɳ_p_*^2^ = 0.35, −10 ± 33%), as well as increased average handgrip strength (*p* = 0.001, *ɳ_p_*^2^ = 0.45, 6 ± 12%), and gait speed (*p* = 0.005, *ɳ_p_*^2^ = 0.27, 0.09 ± 0.16 m/s) in both groups. Interestingly, SBF caused a greater increase in peak handgrip strength (8 ± 14%), compared to LIPA (2 ± 10%; *p* = 0.04, *ɳ_p_*^2^ = 0.38).

**Conclusion:** SB displacement induced significant improvements in fasting triglycerides, gait speed, as-well as STS endurance/speed in older women. Frequent vs. continuous SB displacement also caused greater increases in handgrip strength. While both SB displacement protocols display promise as efficacious home-based interventions for self-isolating older adults, our results would suggest a physical functioning advantage of the SBF protocol for certain outcomes.

## Introduction

The rapid spread of Coronavirus disease 2019 (COVID-19) has prompted many nationwide lockdowns (Lu et al., 2020; Sohrabi et al., 2020). In most cases, it is understood that patients requiring intensive care, are more likely to be older (Wang et al., 2020), prompting the call for all older adults (herein defined as ≥65y), to shield themselves, by proceeding to immediately begin prolonged and strict self-isolation (Armitage and Nellums, 2020). Habitually, older adults spend ~65–80% of their waking hours performing sedentary behavior (SB; Wullems et al., 2016; Loyen et al., 2017). SB is associated with sarcopenic obesity (Henson et al., 2018; Reid et al., 2018), reduced bone mineral density (BMD; Onambele-Pearson et al., 2019), heightened cardio-metabolic risk profile (Biswas et al., 2015; Hadgraft et al., 2020), frailty (da Silva et al., 2019), and premature mortality (Ekelund et al., 2019), in older adults. Furthermore, women tend to exhibit greater anabolic resistance and larger reductions in strength following disuse compared to men (Smith et al., 2008, 2012). Self-isolation is likely to exacerbate SB, given that habitual SB is primarily accumulated at home, during social isolation (Leask et al., 2015; Dontje et al., 2018). Despite acknowledgement of their limited efficacy/palatability (Chen et al., 2020; Jiménez-Pavón et al., 2020; Lippi et al., 2020), the default solution is simply recommending that older adults engage in moderate to vigorous physical activity [structured exercise (moderate to vigorous physical activity, MVPA)] with no clear directives *vis-a-vis* breaking up sitting time. However, many barriers inhibit long-term adherence to conventional MVPA recommendations (≥150 min/week, ~21 min/day; World Health Organization, 2010) in older adults (Hansen et al., 2019), including a lack confidence (Forkan et al., 2006) and appropriate equipment (Rhodes et al., 1999; Forkan et al., 2006; Bell et al., 2007). Given such barriers, older adults report a poor tolerance for intense physical activity, including greater perceived difficulty, and greater dropout rate (Onambélé-Pearson et al., 2010; Brawner et al., 2016). This can be problematic in the long term as only supramaximal MVPA engagement (≥ 420 min/week, ~60 min/day), appears to offset the negative health effects of concurrent high SB time (Ekelund et al., 2016; Manas et al., 2019). Furthermore, given that sudden surges in exercise can compromise immune response (Siedlik et al., 2016; Nieman and Wentz, 2019), reduced protection from infections like COVID-19, is a further concern. Such limitations create scope for safer alternative home-based interventions to mitigate the potential for further compromised health during self-isolation.

Displacing or breaking up SB time is one such viable option. Promisingly, older adults perceive SB displacement as acceptable and easy to incorporate in their daily routine (Matson et al., 2018). Light intensity physical activity (LIPA) during SB displacement, is a pre-requisite for long-term health benefits (Dohrn et al., 2018; Chastin et al., 2019; Stamatakis et al., 2019), due to LIPA generating superior responses in both muscle activity (MA; Tikkanen et al., 2013; Lerma et al., 2016), and energy expenditure (Carter et al., 2015; Lerma et al., 2016; Saeidifard et al., 2018), compared to stationary standing. Acute reductions in both postprandial glucose (Bailey and Locke, 2015; Welch et al., 2019) and triglycerides (TGs; Miyashita et al., 2016; Kashiwabara et al., 2017), as-well as chronic functional improvement (Barone Gibbs et al., 2017; Harvey et al., 2018), following SB displacement further highlights its potential to enhance cardio-metabolic health and physical function in older adults. However, despite a clearly established link with SB (Hamer and Stamatakis, 2013; Aggio et al., 2016), many functional markers like handgrip strength (HGS), have yet to be investigated. Furthermore, previous studies have merely displaced SB in arbitrary fashion without controlling for the prescribed pattern of LIPA. SB tends to be accumulated in prolonged uninterrupted bouts (Schlaff et al., 2017), which are associated with worse health outcomes (Gennuso et al., 2013, 2016; Diaz et al., 2017), compared with a more fragmented pattern. Therefore, a longitudinal intervention trial is warranted to investigate the chronic effects of SB displacement on health in older adults, while elucidating what role the pattern (fragmentation vs. a single bout) of prescribed LIPA plays in benefiting anyone but especially self-isolated frail older adults such as during the COVID-19 pandemic.

Therefore, the aims of this study were to (1) compare the chronic effects of two distinct SB displacement interventions on commonly assessed markers of health in older adults and (2) examine the impact prescribed patterns of activity have on the aforementioned outcomes. Given the clearly established link between SB and poor health outcomes in older adults (da Silva et al., 2019; Ekelund et al., 2019; Hadgraft et al., 2020), especially when SB is accumulated in a prolonged pattern (Gennuso et al., 2013, 2016; Diaz et al., 2017), it was hypothesized that (1) SB displacement would have small yet positive effects on markers of health and physical functioning in older adults and (2) SB fragmentation (SBF) throughout the day would induce greater health benefits compared to a single continuous bout of LIPA.

## Materials and Methods

### Participants and Experimental Design

Twenty-eight elderly women voluntarily participated in the study. Ethical approval was obtained [230118-ESS-DG-(2)], and written informed consent obtained prior to any procedures being performed, in line with the Declaration of Helsinki. Participants were recruited from the local community (Cheshire East) through advertising (posters, speaking engagements, etc.) and from a research volunteer database (local participants). Prior to the general data protection regulation deadline on May 25, 2018, recruitment packages (which included “General Data Protection Regulation” opt in/out permission slips, health questionnaires, participant information sheets, informed consent forms, and a pre-paid return envelope), were sent to all contacts aged 65–85 years. Returned questionnaires were screened for potential eligibility. Exclusion criteria included recent history of lower limb disorders, or current chronic health conditions [e.g., cardiovascular disease (CVD), uncontrolled diabetes, active cancer, etc.], likely to affect their ability to safely and independently undertake a program of decreased SB. Estimation of required sample size to detect significant changes in the desired outcomes was based upon the fact that previous SB interventions in older adults that have observed improvements to physical function, utilized total sample sizes of ~25–38 (Rosenberg et al., 2015; Barone Gibbs et al., 2017; Harvey et al., 2018). The current achieved sample size of 28 older women, falls within this range. Participants underwent familiarization and, after 7 days, returned to the laboratories to undergo body composition analysis, blood sampling, and functional assessments. Participants were then randomly allocated in a 1:1 fashion to one of two groups: (1) SBF (*n* = 14) or (2) single bout LIPA (*n* = 14). All measures were taken at weeks 0 (baseline) and 8 (post intervention).

### Body Composition

A dual energy x-ray absorptiometry (DEXA) scanner (Hologic Discovery: Vertec Scientific Ltd., United Kingdom) was used (whole body procedure, EF 8.4 lSv; Tomlinson et al., 2014), to ascertain BMD, lean body mass (LBM), and body fat percentage (BFP%) metrics.

### Blood Sampling

A 20 ml blood sample was drawn using a 0.5 Inch 23 g BD Needle (Mistry Medical Supplies, England). Whole blood analyses of fasting plasma glucose, total cholesterol, and TGs were performed using an Accutrend Plus (Roche Diagnostics Limited, United Kingdom), while glycated hemoglobin (HbA1C%) was analyzed using a 501 device (HemoCue, Sweden). Accordingly, both Accutrend and Hemocue have shown good reliability (Luley et al., 2000; Newman and Turner, 2005; Phillips et al., 2014) and validity (Luley et al., 2000; Coqueiro et al., 2014; Hirst et al., 2017), when compared to laboratory testing.

### Physical Function Assessment: Gait Speed, Sit-to-Stand Ability, and Handgrip Strength

A modified pressure sensor (Tekescan, United States) and height adjustable stool were used to reduce testing variability (Demura and Yamada, 2007). Gait speed was assessed through the timed up and go test (TUG; Podsiadlo and Richardson, 1991). Participants rose from the chair and walked at maximum speed to a marker 6 m away before returning to the seated position. Gait speed was defined as the quickest speed recorded over three trials [meters per second (m/s)]. Participants were then instructed to rise from the chair until the knee was fully extended and then return to a seated position. This was performed once as quickly as possible in the case of the one sit-to-stand (1STS, functional speed), and as many times as the participant could perform in an exact 30 s time frame for the 30STS (30 s STSs, functional endurance). A handgrip dynamometer (Takei, Japan), was used to assess HGS. Dynamometry is both a reliable and valid measure of strength in the elderly (Bohannon and Schaubert, 2005; Abizanda et al., 2012). Briefly, participants were instructed to maximally squeeze the handle and discontinue grasping at self-perceived maximum voluntary effort. Three trials were performed on each hand, with peak HGS defined as the maximum value achieved across both hands, and the average of three trials used to provide an average of both arms. Importantly, gait speed (Studenski et al., 2011), STS ability (Cooper et al., 2010), and grip strength (Sasaki et al., 2007) are all significant predictors of mortality in older adults.

### Physical Behavior Interventions

The purpose of the two intervention groups was to manipulate the protocol for displacing SB time with added daily LIPA (45–50 min in total). The interventions were confined to a 12-h period between 09:00 and 21:00. The prescribed amount of LIPA (45–50 min) was based upon two key points. First, the WHO’s MVPA recommendation (World Health Organization, 2010) gives a theoretical starting point for what activity amount may be beneficial. Utilizing metabolic equivalent of task (MET) thresholds (SB: <1.5 METs, LIPA: 1.5–3.0 METs, MVPA: >3.0 METs), 150 min/week translates into ~21 min/day moderate activity (~64 MET·min/day), meaning the same amount of MET·min/day, performed in LIPA (with a minimum intensity of 1.6 METs), would theoretically total ~40 min/day. Furthermore, the SBF group was instructed to fragment sitting time every 30 min over a 12-h period (09:00-21:00), based on recent epidemiological evidence linking a more prolonged sedentary accumulation pattern (≥30 min bouts) with greater all-cause mortality (Diaz et al., 2017). Consequently, this totaled a maximum of 24 2-min LIPA bouts throughout the day (48 min). Envisaging a varied compliance response, the LIPA group was prescribed a range for their single continuous bout. Accordingly, the prescribed amount of LIPA (an additional 45–50 min per day), was equally matched between the two groups, whereas the prescribed pattern (intermittent micro-bouts vs. single continuous bout) was different. Both intervention groups were provided with an illustrated booklet, which contained LIPA suggestions compiled from the compendium of physical activities (Ainsworth et al., 2011). Importantly such activities were intentionally selected due to their simplicity, safety, and ease of implementation within the home environment.

Individual participant compliance was objectively monitored at weeks 0 and 8, using a thigh mounted GENEActiv original triaxial accelerometer (Activinsights Ltd., United Kingdom). Data were subsequently extracted using GENEA software, and a previously validated algorithm (Wullems et al., 2017) used for baseline and post-intervention data analysis. Briefly, the aforementioned validation study calculated the incremental metabolic cost of 10 everyday tasks in 40 healthy older adults (~74 years; e.g., lying down, brisk treadmill walking, etc.), and used regression analysis to identify specific physical activity intensity ranges [utilizing MET thresholds (SB: <1.5 METs, LIPA: 1.5–3.0 METs, MVPA: >3.0 METs)] mapped against the concurrently recorded GENEActiv gravitational pull and acceleration data. The robustly derived data on SB, standing, LIPA, and MVPA in older adults were used for further analyses. Participants were also further classified as physically active (≥150 min/week MVPA_≥10 min bouts_), or non-physically active (<150 min/week MVPA_≥10 min bouts_), given that the World Health Organization (WHO) recommends a weekly MVPA engagement time of 150 min/week (World Health Organization, 2010).

#### SBF Group

Participants were told that the purpose of their intervention was to reduce the amount of time spent performing SB (sitting, lying, or reclining) especially in prolonged uninterrupted bouts. Participants were instructed not to perform SB for more than 30 min at a time, and that for every 30 min of SB performed the participant should stand up and perform 2 min of upright LIPA (general ambulatory walking, side to side shuffling, washing dishes, etc.).

#### LIPA Group

Participants were informed that the purpose of their intervention was to increase the amount of time spent performing LIPA while maintaining habitual routines. Participants were instructed to perform a continuous single bout of 45–50 min LIPA (general ambulatory walking, side to side shuffling, washing dishes, etc.), every day for the duration of the 8-week intervention.

### Palatability Assessment

During the post-test visit, participants were asked to complete a palatability questionnaire. Each question was designed to rate an aspect of the participants experience and gain insight on perceived quality of life (QoL).

### Statistical Analyses

Statistical analyses were carried out using SPSS (Version 26, SPSS Inc., Chicago, IL, United States). Normal distribution and equality of variances between groups were checked using the Shapiro-Wilk and Levene’s tests, respectively. Baseline group differences were subsequently examined with an independent sample’s T-test or Mann-Whitney U test (SBF vs. LIPA) as appropriate. The effects of the interventions were determined using 2 × 2 split plot ANOVA [two time phases (pre and post intervention) and two intervention groups]. In cases of non-normally distributed data, within group comparisons were made using the Wilcoxon-Sign Rank test, while, between group differences were analyzed through a Mann-Whitney U test on the relative changes from baseline. A Chi squared test was used to compare between group differences for ordinal/nominal data from the palatability questionnaire. Furthermore, Spearman bivariate correlations were utilized to investigate associations between the relative changes in LBM metrics and the relative changes in functional assessments. Data are reported as mean ± SD [or median ± interquartile range (IQR) for non-parametric data]. Statistical significance was accepted when *p* ≤ 0.05. Furthermore, a statistical trend was deemed to be present when *p* was in the range of between 0.05 and 0.10. Effect size (*ɳ_p_*^2^) was also reported, where *p* is significant.

## Results

### Descriptive Characteristics of Participants at Baseline

The 28 older women (age: 73 ± 5 years, height: 1.60 ± 0.07 m, weight: 67 ± 10 kg, and BMI: 26.1 ± 3.6 kg.m^2^) were matched at baseline for all outcomes of interest (*p* > 0.05), denoting a well-matched study sample (see Table 1).

**Table 1:** Participant characteristics at baseline.

Participants characteristics	SBF (*n* = 14)	LIPA (*n* = 14)
Age (y)	74 ± 5	73 ± 6
Weight (kg)	68.6 ± 11.3	65.5 ± 8.6
BMI (kg.m^2^)	26.9 ± 3.6	25.3 ± 3.6
Total lean body mass (LBM; kg)	39.3 ± 5.7	37.2 ± 3.9
Proportional T-score classification as osteoporotic/osteopenic (normal)	29% (71%)	43% (57%)
Proportion who live alone/(cohabitate)	36% (64%)	43% (57%)
Polypharmacy (*n*)	2 ± 2	1 ± 1
FRAT (number of positive responses)	1 ± 1	1 ± 1

FRAT, falls risk assessment tool; LIPA, light intensity physical activity; and SBF, sedentary behavior fragmentation.

### Intervention, Compliance, and Palatability

**Figure 1:**
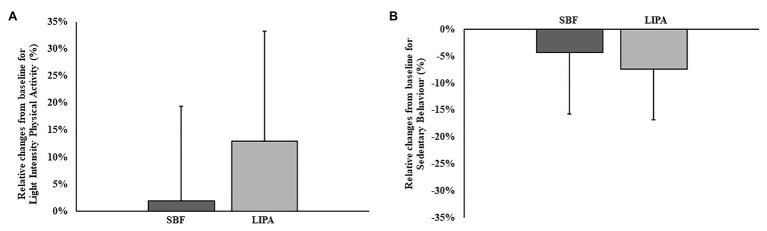
Group dependent changes relative to baseline for physical behaviour outcomes. Panel **A** represents changes in light intensity physical activity (LIPA), while panel **B** represents changes in sedentary behaviour.

No differences existed between groups regarding the number of intervention days (days from pre-lab visit to post) that the participants undertook (SBF: 56 ± 2 days, LIPA:56 ± 1 days; *p* = 0.37). Regarding 3D-accelerometer-based compliance data, both groups were matched for all variables at baseline. SB significantly decreased over time in both groups (*p* = 0.006, *ɳ_p_*^2^ = 0.26), but did not exhibit a group × time interaction (*p* = 0.41; Figure 1). Similarly, mean SB bout time significantly decreased over time in both groups (*p* = 0.045, *ɳ_p_*^2^ = 0.27), but did not exhibit a group × time interaction (*p* = 0.96; Table 2). LIPA significantly increased over time in both groups (*p* = 0.04, *ɳ_p_*^2^ = 0.15), but did not exhibit a group × time interaction (*p* = 0.11; Figure 1). Standing and MVPA time, however, did not significantly change (Table 2). Concerning intervention palatability, promisingly, all participants agreed the instructions were easy to follow at home, with 89% reporting increased awareness of their daily sedentarism. Accordingly, 82% of participants reported feeling more positive about their health, and most importantly, 61% of participants stated they could definitely continue following this intervention long term. Furthermore, 54% of participants stated their intervention had motivated them to become more active. However, only 25% of participants stated they definitely felt more confident about performing household tasks following their respective interventions. There was no difference in self-reported satisfaction or continued adherence between groups (*p* ≥ 0.05).

**Table 2:** Pre and post values for health outcomes.

		SBF (*n* = 14)		LIPA (*n* = 14)	
		Pre	Post	Pre	Post
LBM (dual energy x-ray absorptiometry, DEXA)	Arms (kg)	1.86 ± 0.32	1.85 ± 0.28	1.78 ± 0.21	1.76 ± 0.23
	Legs (kg)	6.18 ± 1.19	6.04 ± 0.95	5.89 ± 0.75	5.84 ± 0.68
Bone mineral density	Thoracic spine (g/cm^3^)	0.90 ± 0.09	0.94 ± 0.13	0.90 ± 0.14	0.94 ± 0.15
	Lumbar spine (g/cm^3^)	0.98 ± 0.16	0.96 ± 0.15	0.97 ± 0.16	0.98 ± 0.16
	Total (g/cm^3^)	1.10 ± 0.11	1.09 ± 0.11	1.11 ± 0.15	1.11 ± 0.14
Adiposity indices	Total (kg)	26.1 ± 6.6	26.5 ± 6.3	25.1 ± 5.5	25.3 ± 5.8
	Android: Gynoid ratio	0.94 ± 0.15	0.93 ± 0.14	0.93 ± 0.18	0.90 ± 0.17
	Waist (cm)	92 ± 18	92 ± 24	91 ± 5	92 ± 8
	Hip (cm)	100 ± 7	99 ± 6^*^	100 ± 8	99 ± 8^*^
	WHR	0.95 ± 0.11	0.95 ± 0.18	0.91 ± 0.10	0.92 ± 0.10
	Body fat percentage (BFP, %)	39 ± 7	38 ± 5	38 ± 7	38 ± 7
Cardio-metabolic biomarkers	HBA1C (%)	5 ± 1	6 ± 0	6 ± 1	6 ± 1
	Glucose (mmol/L)	5.34 ± 0.98	5.01 ± 1.73	4.94 ± 0.86	4.73 ± 0.88
	Triglycerides (mmol/L)	2.19 ± 0.82	1.94 ± 0.50^*^	1.94 ± 0.52	1.68 ± 0.40^*^
	Total cholesterol (mmol/L)	5.53 ± 1.47	5.80 ± 1.86	5.33 ± 1.58	5.97 ± 1.25
Physical function	Peak HGS (kg)	26.3 ± 8.5	26.8 ± 6.1^*^^×^	26.5 ± 4.4	26.5 ± 7.3^*^^×^
	Average HGS (kg)	22.8 ± 6.6	23.9 ± 5.4^*^	22.9 ± 5.7	23.8 ± 7.2^*^
	30STS	14 ± 3	17 ± 3^*^	17 ± 3	18 ± 4^*^
	1STS (s)	2.49 ± 1.02	2.15 ± 0.70^*^	1.98 ± 0.52	1.86 ± 0.52^*^
	Max gait speed (m/s)	1.17 ± 0.22	1.26 ± 0.19^*^	1.22 ± 0.13	1.28 ± 0.11^*^
Daily physical behavior	SB time (hr/24 h)	9.6 ± 1.2	9.2 ± 1.6^*^	9.6 ± 1.1	8.9 ± 1.2^*^
	Standing time (hrs/24 h)	1.0 ± 0.6	1.0 ± 0.6	1.4 ± 1.1	1.5 ± 0.7
	LIPA time (hrs/24 h)	2.2 ± 0.5	2.2 ± 0.6^*^	2.1 ± 0.4	2.3 ± 0.5^*^
	MVPA time (hrs/24 h)	3.0 ± 1.0	2.8 ± 1.0	2.5 ± 0.8	2.8 ± 0.7
	Mean SB bout time (min)	31 ± 8	27 ± 9^*^	32 ± 14	29 ± 11^*^
	Proportion meeting recommended MVPA time [≥150 min/week MVPA (≥10 min bouts; World Health Organization, 2010)]/ below recommended MVPA	29%/71%	7%/93%	0%/100%	7%/93%

* Significant change from baseline.

× Significant group dependent effect.

HGS, handgrip strength; LIPA, light intensity physical activity; MVPA, moderate to vigorous physical activity; SB, sedentary behavior; SBF, sedentary behavior fragmentation; STS, sit-to-stands; and WHR, waist to hip ratio.

### Bone Mineral Density

After accounting for previously identified co-variates [total body fat (TFAT), Android:Gynoid fat ratio (AGR), and BMI; Onambele-Pearson et al., 2019], thoracic (*p* = 0.09, *ɳ_p_*^2^ = 0.12), but not lumbar spine mineral density (*p* = 0.70), exhibited a trend to change over time. Importantly, thoracic spine did not exhibit a significant group × time interaction effect (*p* = 0.71) with changes similar in both groups (SBF: 5 ± 14%, LIPA: 4 ± 9%).

### Body Composition

**Figure 2:**
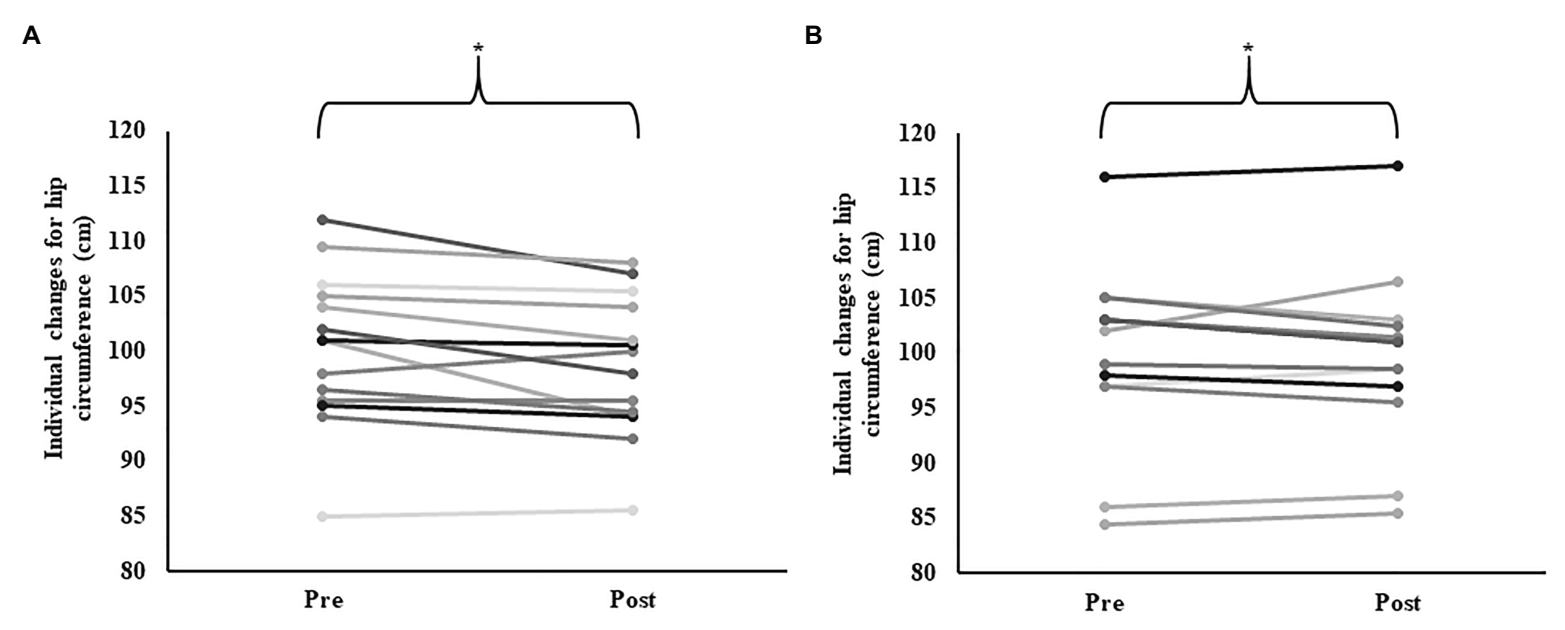
Group dependent individual changes for hip circumference. Panel **A** represents individual changes following sedentary behaviour fragmentation (SBF); while panel **B** represents individual changes following continuous light intensity physical activity (LIPA). *Represents a significant change over time.

Neither arm (*p* = 0.73), leg (*p* = 0.17), nor total (*p* = 0.20) LBM, significantly changed over time. Despite no change in BFP% (*p* = 0.12), we did observe trends for AGR (*p* = 0.08, *ɳ_p_*^2^ = 0.11), and TFAT (*p* = 0.10, *ɳ_p_*^2^ = 0.004), to change over time (Table 2). We also observed a significant reduction in hip circumference over time (*p* = 0.02, *ɳ_p_*^2^ = 0.19), as-well as a trend (*p* = 0.07, *ɳ_p_*^2^ = 0.12), toward a group × time interaction effect (Table 2) primarily driven through apparent greater reductions in SBF (−1.79 ± 2.34 cm), compared to LIPA (−0.25 ± 1.94 cm; Figure 2).

### Cardio-Metabolic Biomarkers

**Figure 3:**
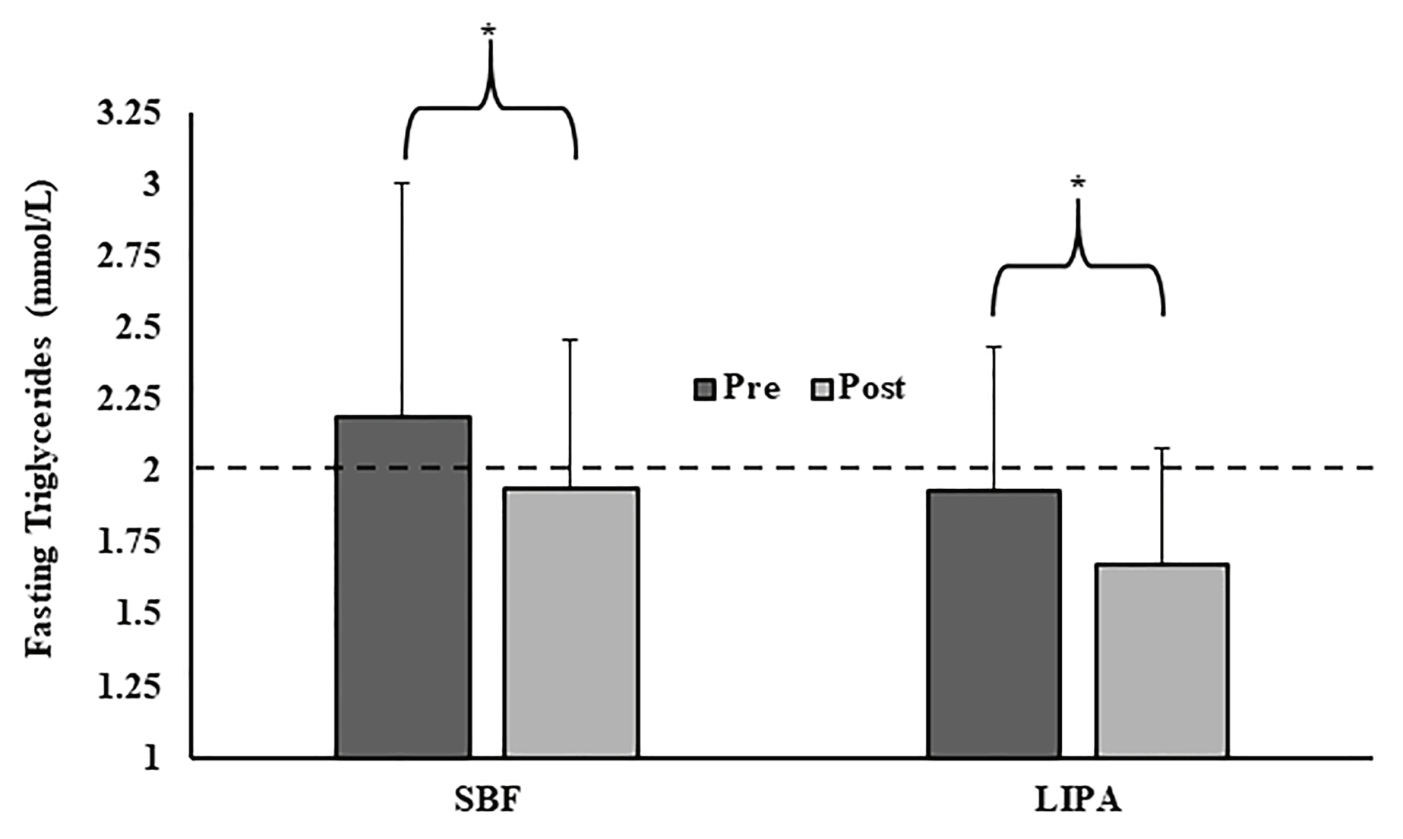
Group changes in fasting triglycerides from pre to post intervention. *Represents a significant change over time. The dashed line (- - -) represents the fasting triglycerides threshold (2 mmol/L) below which significantly reduced CVD risk is observed (Iso et al., 2014; Nordestgaard and Varbo, 2014).

We observed a significant main effect of time for fasting blood TG (*p* = 0.045, *ɳ_p_*^2^ = 0.15), which was similar in the two groups, given no significant group × time interaction (*p* = 0.98; SBF: −0.26 ± 0.77 mmol/L, LIPA: −0.26 ± 0.51 mmol/L; Figure 3). No other cardio-metabolic serum biomarkers exhibited main effects for group, time, or group × time interactions.

### Physical Function

**Figure 4:**
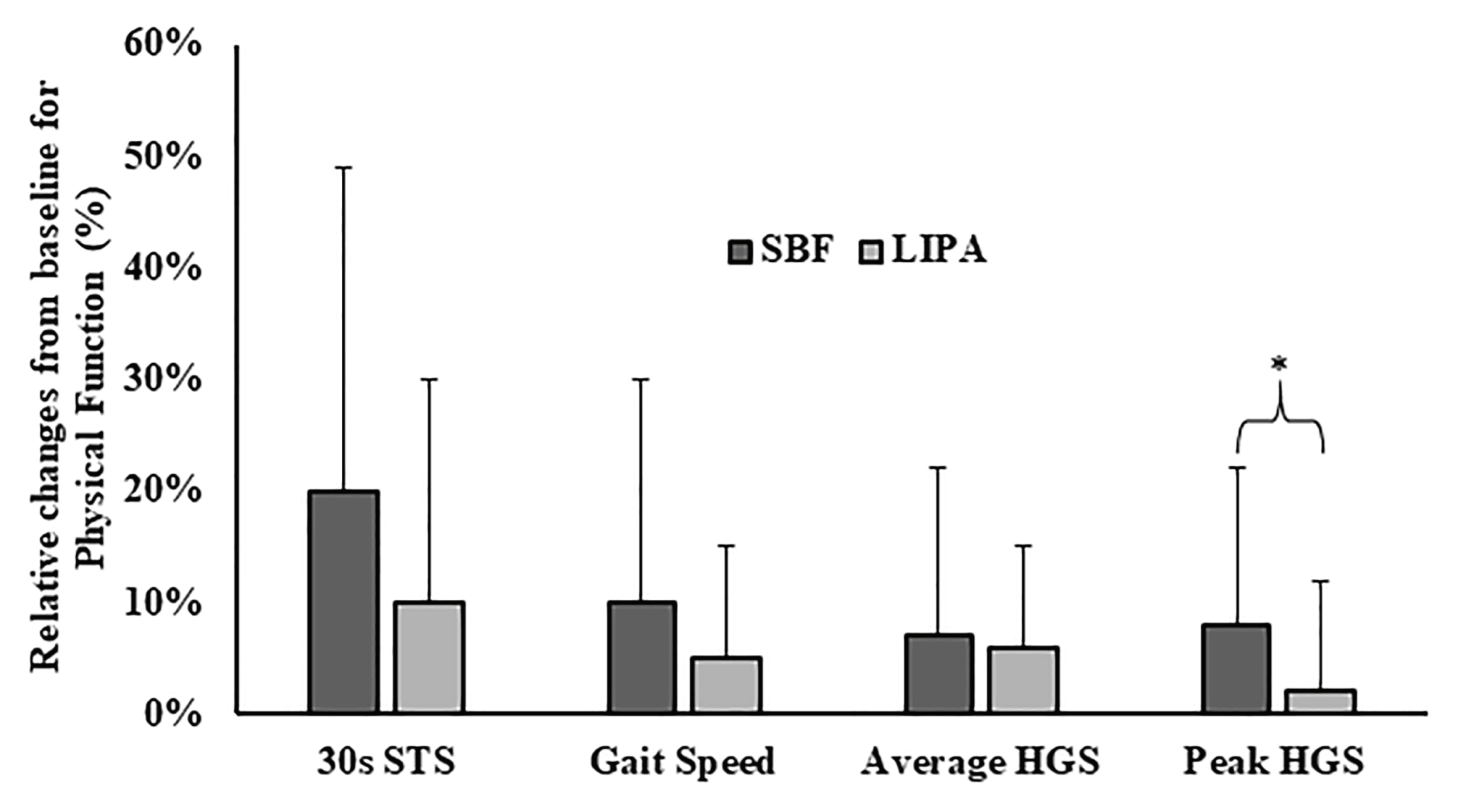
Group dependent changes relative to baseline for physical function parameters. *Represents a significant group × time interaction effect. HGS, handgrip strength and STS, sit-to-stands.

A significant main effect for time was exhibited for gait speed (*p* = 0.005, *ɳ_p_*^2^ = 0.27, 0.09 ± 0.16 m/s), but not a group× time interaction effect (*p* = 0.44). There was also a significant main effect of time for 30STS (*p* = 0.002, *ɳ_p_*^2^ = 0.32, 2 ± 3 STS), 1STS (*p* = 0.009, *ɳ_p_*^2^ = 0.35, −10 ± 33%; see Figure 4), and average HGS (*p* = 0.001, *ɳ_p_*^2^ = 0.45, 6 ± 12%). Furthermore, peak HGS was the only functional outcome to exhibit both a significant main effect of time (*p* = 0.044, *ɳ_p_*^2^ = 0.27), and a group × time interaction (*p* = 0.04, *ɳ_p_*^2^ = 0.38), with a greater increase in SBF than LIPA (SBF: 8 ± 14% and LIPA: 2 ± 10%; Figure 4). Interestingly, the relative change from baseline in arm LBM, was significantly associated with the relative change from baseline in peak HGS (*R*^2^ = 0.17 *p* = 0.03), accounting for ~17% of the explained variance when both groups were pooled. Furthermore, when sub-analyzed by group, such an association persisted in LIPA (*R*^2^ = 0.53, *p* = 0.004) but not SBF, accounting for 53% of the explained variance.

## Discussion

This is the first study to investigate the chronic effects of SB displacement on health outcomes in older women and provide recommendations to mitigate any negative health consequences. We hypothesized that SB displacement would have measurable and positive effects on markers of health and physical functioning in older adults. We observed significant improvements over time for circulating TG, hip circumference, gait speed, 30STS, 1STS time, average HGS, and peak HGS, thereby upholding our first hypothesis. We further hypothesized that SBF would induce greater benefits compared to continuous LIPA. Here, we observed a trend for hip circumference (*p* = 0.07) and a significant effect for peak HGS (*p* = 0.04) to exhibit the predicted SBF advantage. Consequently, the second hypothesis was partially upheld.

We observed significant functional improvements post-intervention. Firstly, increased muscular endurance (30STS) and enhanced gait speed are consistent findings across previous SB studies (Barone Gibbs et al., 2017; Harvey et al., 2018), potentially highlighting a specificity of training effect, improving one’s ability to mobilize from a seated position. We also observed for the first time a decrease in the time taken to complete 1STS (an index of functional speed), further suggesting improved movement execution and enhanced muscular power. This positive effect is of notable impact given that inappropriate STS transitions are responsible for up to 41% of falls in care home residents (Rapp et al., 2012). Importantly, peak HGS improved to a greater extent in SBF compared to LIPA. Holding onto the arm of a chair and pushing through one’s arms are common cues given to older adults when performing STS (Kindblom-Rising et al., 2010). Therefore, we propose that the increased STS frequency may have also increased the frequency with which the SBF participants utilized the arm stabilization tactic, subsequently, causing gradual functional adaptation in the upper body (including arm) musculature. Nevertheless, we advised all participants to implement many upright upper body tasks (sweeping up, etc.), and improvements in HGS have been reported following implementation of light upper body based movements (Nicholson et al., 1997; Anthony et al., 2013; Sexton and Taylor, 2019). Interestingly, arm LBM did not significantly change from pre to post, yet the relative change in arm LBM significantly accounted for 17–53% of the explained variance for the change in peak HGS. The greater association between muscle tissue content and peak HGS in the LIPA group may be linked to the fact that these participants were requested to perform various operational tasks in a continuous fashion, which appears to have caused a statistically insignificant yet clinically meaningful hypertrophic response leading to enhanced peak HGS. The observed improvements in lower body muscular endurance/power, in both intervention groups, as-well as HGS are compelling positive changes associated with an exercise intensity not customarily regarded as optimal.

We observed significant reductions in fasting circulating TG. Acutely interrupting sitting time with brief bouts of LIPA attenuates postprandial TG concentrations (Miyashita et al., 2016; Kashiwabara et al., 2017), and habitual LIPA is associated with reduced TG in older adults (Ryan et al., 2015), which in turn is linked to reduced CVD risk (Baigent et al., 2010). Our data demonstrate such acute effects, persist into accumulated long-term benefits. Importantly, fasting TG levels below 2 mmol/L, confer significantly reduced risk of CVD (Iso et al., 2014; Nordestgaard and Varbo, 2014), a level that was beneficially achieved by both groups, post intervention (SBF: 1.94 ± 0.50 mmol/L and LIPA: 1.68 ± 0.40 mmol/L). Increased lipoprotein lipase (LPL) is a probable underlying mechanism, given the significant role it plays in reduced CVD risk (Hamilton et al., 2007). We thus propose persistent increases in the energy demand of contracting muscle facilitated enhanced substrate uptake. In contrast to previous evidence showing a more fragmented SB pattern is associated with decreased TG (Carson et al., 2014; Brocklebank et al., 2015), our data suggest the prescribed LIPA pattern is not of such relative importance, given that both groups decreased TG to a similar extent over time. Elucidating alterations in peripheral insulin sensitivity, requires a glucose tolerance test (Davies et al., 2000; Petersen and McGuire, 2005; Tabák et al., 2012), which we recommend future studies investigate. As they currently stand, our data simply suggest that chronic SB displacement in older women causes beneficial reductions in fasting circulating TG, irrespective of prescribed pattern.

Our data show reduced hip circumference following both interventions. Given that LIPA raises energy expenditure (Carter et al., 2015; Lerma et al., 2016), a chronically sustained LIPA increase likely created a negative energy balance (Levine et al., 2005), beneficially reducing what we will assume were fat deposits around the hips. Furthermore, AGR also exhibited a trend to decrease over time. Together, these changes can be viewed as positive given that abdominal adiposity (Android) is more detrimental to health compared to lower body accumulation (Bastien et al., 2014; Chrysant and Chrysant, 2019). In support of our findings, a previous exercise intervention has noted a reduction in waist to hip ratio (de Mendonça et al., 2014). We also observed trends toward improved thoracic spine BMD. Accordingly, LIPA is associated with increased thoracic spine BMD in older adults (Onambele-Pearson et al., 2019), where the authors speculated excessive kyphotic curvature likely increases (forward) shear forces between thoracic vertebrae while walking (Kohrt et al., 1997), and thus places stress/strain on the bone structures, sufficient to cause adaptation. Our findings therefore support the notion of beneficial body composition changes (statistically significant and trends) following SB displacement in older women.

Together with the successful implementation of a randomized chronic SB intervention study in older adults, the novel manipulation of the prescribed LIPA pattern, makes the current study’s design one of its primary strengths. Despite the lack of a control group limiting our design, the different patterns of LIPA prescription took priority. Furthermore, while the exclusive inclusion of older women somewhat limits the generalizability of our findings, we ultimately see this as a strength, given that muscle-tendon adaptation to resistance training appears to be gender dependent (McMahon et al., 2018). Moreover, we collected data on a range of health and physical functioning markers, 3D-accelerometer-based compliance, and self-reported adherence following SB displacement. Given that we successfully altered objectively measured SB, LIPA, and SB bout length in our participants, this reinforces our conclusion, that SB displacement specifically with LIPA mediated the health improvements observed. Furthermore, both interventions, were similarly rated as easy to implement at home, increased awareness of habitual SB, self-perceived health, and marked likelihood to integrate into lifestyle in the long term. Our findings add to the knowledge-base in the topic of SB effects (Wu et al., 2013; Matson et al., 2018, 2019; Wilson et al., 2019). Perhaps future studies could implement a similar intervention strategy, while elucidating the physiological mechanisms that underpin such positive changes, including muscle-tendon complex adaptation (e.g., neuromuscular adaptation), serum lipid transporters (e.g., LPL), and biological markers of inflammation (interleukin 6, tumor necrosis factor alpha, and C-reactive protein). Future studies should also investigate the effects of SB displacement on validated QoL assessments in older adults (e.g., SF-36 and EQ-5D; Bohannon and DePasquale, 2010), as-well as comprehensive physical capacity assessments [e.g., 6 min walk test (6MWT; Agarwala and Salzman, 2020)].

Given that older adults are being requested to “shield” and engage in prolonged and strict self-isolation (Armitage and Nellums, 2020), this makes the results of the current study very applicable. Accordingly, we recruited community dwelling older women from the local community, the population in need of targeted activity interventions during COVID-19 related quarantine. Our results suggest displacing SB with LIPA enhances various markers of health status. Such an intervention can be carried out from the home environment, with minimal effort/support, and displays good likelihood of long-term compliance. However, it must be noted, participants received a fortnightly home visit from the principal investigator to facilitate compliance and troubleshoot issues, which under quarantine conditions is simply not permitted. Such a limitation could be somewhat mitigated through indirect means of contact (telephone calls, emails, video conferencing software, etc.). Nevertheless, our results suggest SB displacement with LIPA is an efficacious home-based intervention for self-isolating community dwelling older adults to mitigate the detrimental health consequences of prolonged sedentarism during quarantine.

## Conclusion

Due to the unrelenting global spread of pandemics such as COVID-19, further quarantine periods are looking increasingly likely for the general population but especially for frail older adults (Lu et al., 2020; Sohrabi et al., 2020). Following 8 weeks of SB displacement with LIPA, we observed significant improvements in blood biomarkers (fasting TGs), and markers of physical function (gait speed, STS endurance/speed, and hand grip strength) in older women. Frequent vs. continuous SB displacement also caused greater increases in peak HGS. Therefore, based on our results, we propose SB displacement is an efficacious home-based intervention for self-isolating older adults, where MVPA is especially challenging. Our data suggest that MVPA engagement is not always necessary for mitigating the detrimental health consequences of prolonged SB. We propose that the positive palatability and high adherence results from our LIPA interventions are testament to the potential for long-term and wide adoption of this type of exercise interventions by key end-users. Furthermore, certain outcomes may be enhanced favorably with fragmented physical activity throughout the day rather than a single bout of exercise, even though both do enhance markers of health and physical functioning.

## Data Availability Statement

The raw data supporting the conclusions of this article will be made available by the authors, without undue reservation.

## Ethics Statement

The studies involving human participants were reviewed and approved by Ethical committee of the Manchester Metropolitan University. The patients/participants provided their written informed consent to participate in this study.

## Author Contributions

GO-P, DT, and KT designed the research. DG conducted the research. DG and GO-P analyzed data, wrote the paper, and had primary responsibility for final content. All authors contributed to the article and approved the submitted version.

### Conflict of Interest

The authors declare that the research was conducted in the absence of any commercial or financial relationships that could be construed as a potential conflict of interest.
